# Germination and First Stages of Growth in Drought, Salinity, and Cold Stress Conditions of Plasma-Treated Barley Seeds

**DOI:** 10.1021/acsagscitech.3c00121

**Published:** 2023-09-06

**Authors:** Alvaro Perea-Brenes, Jose Luis Garcia, Manuel Cantos, Jose Cotrino, Agustín R. Gonzalez-Elipe, Ana Gomez-Ramirez, Carmen Lopez-Santos

**Affiliations:** † Nanotechnology on Surfaces and Plasma Laboratory, Institute of Materials Science of Seville, Consejo Superior de Investigaciones Científicas-Universidad de Sevilla, Seville 41092, Spain; ‡ Department of Plant Biotechnology, Institute of Natural Resources and Agrobiology of Seville, Consejo Superior de Investigaciones Científicas, Seville 41012, Spain; § Departamento de Física Atómica, Molecular y Nuclear, Universidad de Sevilla, Seville 41012, Spain; ∥ Departamento de Física Aplicada I, Escuela Politécnica Superior, Universidad de Sevilla, Seville 41011, Spain

**Keywords:** barley germination, plasma treatment, drought, salinity, proline, photosynthetic pigments

## Abstract

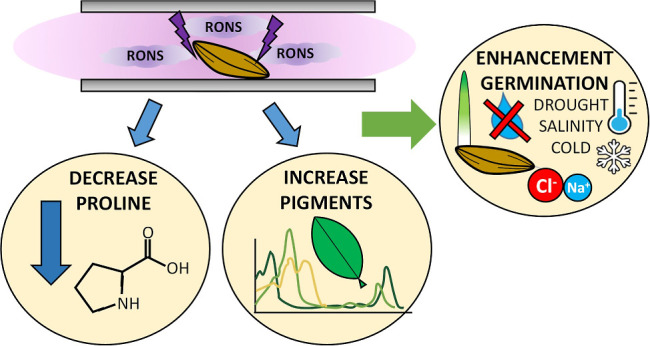

Numerous works have demonstrated that cold plasma treatments constitute an effective procedure to accelerate seed germination under nonstress conditions. Evidence also exists about a positive effect of plasmas for germination under environmental stress conditions. For barley seeds, this work studies the influence of cold plasma treatments on the germination rate and initial stages of plant growth in common stress environments, such as drought, salinity, and low-temperature conditions. As a general result, it has been found that the germination rate was higher for plasma-treated than for untreated seeds. Plasma also induced favorable changes in plant and radicle dimensions, which depended on the environment. The obtained results demonstrate that plasma affects the biochemical metabolic chains of seeds and plants, resulting in changes in the concentration of biochemical growing factors, a faster germination, and an initially more robust plant growth, even under stress conditions. These changes in phenotype are accompanied by differences in the concentration of biomarkers such as photosynthetic pigments (chlorophylls *a* and *b* and carotenoids), reactive oxygen species, and, particularly, the amino acid proline in the leaves of young plants, with changes that depend on environmental conditions and the application of a plasma treatment. This supports the idea that, rather than an increase in seed water imbibition capacity, there are clear beneficial effects on seedling of plasma treatments.

## 1 Introduction

Seed germination is a critical step for the development of plants and, consequently, a process that has been studied from multiple perspectives, including morphological, biochemical, and gene expression.^1^ During the last years, among the large variety of methods utilized to improve seedling and plant growth, the application of low-temperature plasmas has received considerable attention in the scientific literature.^2–9^ Recent reviews on the subject have highlighted the complexity of the biochemical and gene expression effects that can be triggered when treating seeds with plasmas. Also, the diversity of scenarios and variables that must be considered in these studies, including type of plants, characteristics of cold plasmas, or environmental growth conditions.^10–14^

Plasmas have been also applied to improve the water absorption capacity of seeds, avoiding seed dormancy effects and seed borne diseases and increasing seed resistance toward abiotic stresses.^13^ In this regard, very often, the improvements in germination rate^2–7,9^ and plant growth have been linked to an increase in seeds’ water uptake capacity upon their exposure to plasmas.^5,6,15–17^ It has been also highlighted that plasma generation of reactive oxygen species (ROS) and reactive nitrogen species (RNS) (in general RONS) affects the biochemistry, the enzymatic activity, or even the gene expression processes of seeds and plants.^10–14,18–22^ However, the role of ROS or RNS and even their chemical nature is not yet completely clarified or understood, mainly because their short lifetimes make their detection difficult.^23^ Peroxo- and superoxide-like, H_2_O_2_, and NO_*x*_ species have been claimed as possible chemical species, which formed on the surface of plasma-treated seeds and then diffused to their interior, contributing to trigger a series of biochemical and gene regulation processes crucial for the germination and the development of plants. Among others, these processes may produce an increase in the activity of antioxidant enzymes,^10,11,13,24,25^ as well as osmotic adjustment substances (e.g., proline, soluble sugar^24^) as well as a modification in the level of signaling phytohormones such as the growing factor abscisic acid.^12,13,24^ For example, in a recent work of our group on plasma-treated barley seeds, we have shown the connection existing between germination rate and the changes in the content of abscisic acid, likely induced by plasma-generated ROS.^26^ In addition, we also found that plasma induced a certain segregation of K^+^ ions to the surface of seeds and the surface enrichment in various types of nitrogen species including NO_*x*_. However, unlike the rather general consensus that plasma contributes to increase the water absorption capacity of seeds,^5,6,15,24,27^ in this previous work, no significant differences in water imbibition was found between plasma-treated and control barley seeds.

A classical topic by the investigation of seed germination and plant growth is the affectation of these processes when sowing in stress conditions such as drought, hot, or saline environments.^28–35^ In general, most works dealing with the plasma treatment of seeds have been done in laboratory and greenhouse environments under well-controlled conditions of humidity, light irradiation, and temperature (generally fixed temperatures of around 20 °C). In the last years, however, more reports have also accounted for the germination of plasma-treated seeds under drought,^36–40^ heat shock,^20^ salinity,^18,20^ toxic heavy metals,^10,41^ and other stress conditions.^14^ In practically all cases, plasma partially counterbalanced the negative effects of the environment and produced an improvement in germination rate, phenotype characteristics, or physiological activity (e.g., concentration of photosynthetic pigments in leaves), features that were associated with some of the biochemical and gene regulation processes mentioned before.

In this work, we study the effect of plasma on the germination of barley seeds under stress conditions. As a first objective, we aim to verify whether, besides the beneficial effect observed in our previous work under favorable growth conditions,^26^ plasma may also improve the germination rate in drought, salinity, and cold conditions. Second, owing to the relevance usually attributed to the uptake of water in plasma-treated seeds,^12,15,26,27^ we want to determine whether water imbibition increases for plasma-treated barley seeds sown in stress environments. Third, to link germination and plant growth improvements with metabolic changes, we also study the impact of plasma on some physiological functions in plant development. For this purpose, we have determined the concentration of biochemical markers such as photosynthetic pigments (chlorophyll *a* and *b*, carotenoids) and proline phytohormone. These pigments are relevant to adjust the response of plants toward stress environments and their relative concentration in leaves may vary in response to drought, salinity, or warm conditions.^28,42–50^ The amino acid proline is an endogenous phytohormone known for its functions as osmotic regulator and membrane protection factor, functions that are of much relevance when seed germination and plant growth occur in drought and salinity conditions.^51–57^ Herein, the analysis of these biomarkers in the leaves of young plants has served to prove that plasma metabolic changes are responsible for the improvement of germination rate and the state of plants in their initial stages of growth. In addition, using a luminescent reagent, we have determined the differences in ROS concentration existing in the control and plasma-treated seeds. In agreement with other authors,^10,21,22,26^ the obtained results support the hypothesis that plasma-generated RONS may affect the seed internal metabolism, contributing to improve the germination rate and the growth of barley plants, even under harsh conditions. From a practical point of view regarding the sowing of barley in soils and environments that may not be the optimal ones for the plant development, the most relevant finding of this work is that plasma may partially counterbalance the adverse effects of the environment, at least during the initial stages of plant growth.

## 2 Materials and Methods

### 2.1 Seeds and Germination Rate

Barley seeds (*Hordeum vulgare* L.) of planet variety were supplied by Intermalta SA. Owing to its considerable germination success, high productivity, as well as good quality, this variety is used for two rowed and spring cycles as well as for malting processes. The seeds were kept in the dark in a closed plastic bag under ambient conditions of the laboratory.

For the purpose of this work, we define as “germination rate” the percentage of seeds that have germinated at a given time after sowing. Germination rate varied with the environment, whether plants have been subjected or not to plasma treatments, and depending on their germination in soil or in Petri dishes. In soil, a seed is considered germinated when first signs of a plant start to appear on the surface of pots. In a Petri dish, germination is confirmed upon appearance of the radicle.

### 2.2 Plasma Treatment

Plasma treatments were carried out in air at ambient atmospheric pressure (i.e., around 700 mbar) in a parallel plate dielectric barrier discharge reactor. The stainless-steel electrodes (8 cm diameter) were covered with two quartz plates (0.5 mm thickness and 10 cm diameter to avoid edge discharges). The gap between the electrodes was fixed at 4.2 cm. The seeds (usually 35 specimens for each treatment) were placed on the bottom electrode (grounded electrode). The active electrode (top electrode) was activated by high voltage with a TREK (model PD05034) amplifier that was connected to a function generator (Stanford Research System, model DS345). *V*(*t*) and *I*(*t*) signals were recorded with an oscilloscope (TEKTRONIK, model TDS2001C) with a bandwidth of 50 MHz and a sample rate per channel of 500 ms/s. A 1:1000 high voltage probe and a current probe coil (conversion factor of 0.05 V mA^–1^) were used for the recording of *I*(*t*) and *V*(*t*) curves. Figure S1 in the Supporting Information shows that the sinusoidal high voltage signal utilized for the experiments has a frequency of 1 kHz and an amplitude of 8.6 kV. *I*(*t*) amplitude was 6.5 mA and the discharge power 5.3 W calculated from the area of the Lissajous curves.^4^ A flow of ambient air was used as plasma gas. A treatment time of 3 min was taken as standard. In previous works, this time maximized the germination rate in well-controlled laboratory conditions.^26^ Further details about reactor architecture, operating conditions, and species detected in the plasma can be found in refs (4) and (58).

### 2.3 Seedling and Germination in Soil

Plasma-treated and pristine (control) seeds were sown in soil. To determine germination rates, seeds were considered germinated when the aerial part of the plant had just emerged from the substrate. Plant development under the tested stresses was compared with that of seedlings resulting from normal culture conditions. Stress conditions were selected for experiments denoted as drought (water deficit), cold (low temperatures), and salinity (high NaCl content in water). The height of the stalks in drought, salinity, and normal conditions was measured after 7 days from the planting day. Since plants had not evolved after the seventh day from sowing in cold conditions, the height of stalks was measured after 10 days. The following specific sowing conditions were utilized.

#### 2.3.1 Normal (Nonstressed) Conditions

Germination was carried out in alveoli of horticultural cell trashes with a peat substrate. They were placed in an adaptation chamber with 111 μE m^–2^ s^–1^ of flux density of photosynthetic photons (PPFD), 16 h of photoperiod, and an ambient temperature of 24 °C with 80% relative humidity. Alveoli were irrigated with tap water 3 times per week with 5 mL/seed each time.

#### 2.3.2 Drought Conditions

Conditions were similar to those for the normal scenario except for the irrigation doses, which consisted of a single weekly irrigation of 1 mL/seed/alveolus.

#### 2.3.3 Cold Conditions

Germination tests in pots with peat were carried out in a special culture chamber located at CITIUS (University of Seville) serving as greenhouse with 29 μmol·m^–2^ s^–1^ of PPFD, 16 h of photoperiod, at a temperature and relative humidity of 5.1 °C and 65%, respectively. Irrigation with 50 mL (5 mL/seed for 10 seeds/pot) was applied three times per week.

#### 2.3.4 Salinity Conditions

Germination in substrate pots occurred applying 0.2 M NaCl saline irrigation. Germination in peat pots was carried out in the same adaptation chamber and conditions as for normal and drought conditions, applying two irrigations with salty water and one irrigation with normal water each week, the last one to avoid an excessive accumulation of salt in the soil. Each irrigation amounted to 5 mL/seed.

#### 2.3.5 Salinity Conditions and Exogenous Proline

Due to the beneficial effects of proline for the development of plants under stress conditions, this amino acid can be added directly to the leaves or the soil during irrigation.^52–54^ An experiment of that kind has been carried out in our work incorporating proline during watering. Growing conditions and irrigation regime (3 irrigations per week) were similar for salinity conditions, except for the characteristics of the water solution used for irrigation: 0.2 M NaCl + 10 mM proline solution twice a week and 10 mM proline solution once a week. Each irrigation amounted to 5 mL/seed/alveolus.

### 2.4 Germination in Petri Dishes

Germination tests in Petri dishes were done for normal, cold, and salinity conditions. Sets of pristine or plasma-treated seeds were placed on a double Whatman filter paper located in a Petri dish of 9 cm diameter. Watering with 4 mL of Milli-Q water was applied to each dish. For salinity conditions, a 0.2 M NaCl water solution was used. Petri dishes were placed in the dark at either 20 °C (normal and salinity conditions) or 5–6 °C (cold conditions). After successive 24 h periods, the Petri dishes were carefully inspected to identify seeds that might have germinated. A seed was considered “germinated” when the radicle had traversed the seed cover or upon the emergence of the coleoptile. Germinated seeds were counted and placed in another Petri dish.

### 2.5 Water Uptake Experiments

Water uptake experiments were carried out for normal, salinity, and cold conditions. The water absorption capacity was followed at 24 °C using a 0.2 M NaCl saline solution (salinity conditions) or Milli-Q water (normal conditions) and at 5 °C and Milli-Q water (cold conditions). Seeds were immersed in the liquids for progressively longer periods of time and then weighed to determine the percentage of weight increase. Before the weight was determined, the seeds were allowed to dry on a filter paper in air for 1 min.

### 2.6 Pigments and Proline Determination

The method used to determine pigment concentrations has been taken from Lichtenthaler.^59^ In short, 50 mg of fresh plant leaves was frozen in liquid nitrogen to facilitate their crushing with a mortar. Then, 10 mL of 80% acetone solution (v/v) was added to the grinding. The resulting sample was centrifuged at 6000 rcf and 4 °C for 10 min, and then 2 mL of 80% acetone (v/v) was additionally added to the supernatant. The absorbance of the liquid was measured at wavelengths of 663 (A_663_), 646 (A_646_), and 470 nm (A_470_). Following ref (59), intensities were converted into contents of chlorophyll *a* (chl a), b (chl b), and carotenoids (car), expressed in μg of pigment/g fresh leaves weight
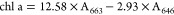
1
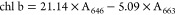
2
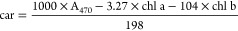
3

The proline content in the leaves was determined applying the method reported in refs (60) and (61). One gram of leaves was ground in liquid nitrogen and then mixed with 5 mL of ethanol. The resulting suspension was then centrifuged for 10 min at 3500 rpm. Subsequently, the supernatant was decanted into a new container. 1 mL of the extract was mixed with 9 mL of distilled water and 5 mL of reagent ninhydrin solution (1.25 g of ninhydrin mixed with 30 mL of glacial acetic acid and 20 mL of 6 M phosphoric acid). The resulting sample was incubated at 65 °C for 45 min in a water bath. After cooling, the absorbance was measured at 515 nm.^60^ The concentration of proline was expressed in milligrams per gram of fresh leaves. Values were deduced comparing the results from experiments with a calibration curve (see Supporting Information Figure S2).

### 2.7 Determination of ROS

Peroxo- and superoxide-like, -H_2_O_2_, content in the seeds was estimated following the colorimetric procedure described by Soares et al.^62^ According to this protocol, peroxo-like species formed or incorporated into the seeds can be extracted through the following steps: (i) grinding 1 g of seeds in 10 mL of phosphate-buffered solution (50 mmol L^–1^, pH 6.5) with 1 mmol L^–1^ hydroxylamine; (ii) centrifugation of the slurry at 6.000*g*_*n*_ for 25 min; (iii) mixing of 3 mL of the separated liquid with 1 mL of titanium sulfate solution at 0.1% in a volume ratio of 20%; (iv) centrifugation of this mixture at 6.000*g*_*n*_ for 25 min; and (v) optical analysis with a spectrometer of the separated liquid. The content of peroxo-like species through the absorption spectra recorded from 200 to 800 nm wavelength for liquids extracted from seeds subjected to various treatments was taken proportional to the absorbance at 410 nm according to the Lambert–Beer law, considering an extinction coefficient of 0.28 μmol^–1^ cm^–1^ and a cuvette path length of 1 cm. Owing to the short lifetime of ROS species at the surface, stress conditions were simulated in the following way: we have fixed 3 min under 0.2 M NaCl solution for salinity stress, 5 min in a fridge at −20 °C for cold stress, and 5 min in a hermetic dry (N_2_ atmosphere) chamber for drought stress. Obtained data correspond to the mean ± SE (standard error) of 5 repetitions for each stress.

### 2.8 Evaluation of Stalks and Roots

To characterize the phenotype of young plants for the different studied situations, the height of their stalks as well as the number of roots and corresponding root length, diameter, and average surface were determined after 10 or 15 days of seedling appearance, depending on whether the stress was due to drought and salinity or cold conditions. These parameters have been estimated by image processing ImageJ software. The calculated covered surface corresponds to the total area occupied by the roots per plant in the corresponding photograph. Resulting values correspond to mean ± SE of 12, 14, and 40 specimens for control and plasma-treated seeds under drought, salinity, and cold stresses, respectively.

### 2.9 Statistical and Sampling Considerations

Results for germination rate in Petri dishes and water uptake experiments (both under cold and salinity conditions) were taken from three replicates with 50 seeds. This renders a total of 150 seeds per treatment and test. Data for germination rate in soil under drought conditions resulted from two replicates with 20 seeds (40 seeds for treatment); in peat under salinity conditions, data corresponded to six replicates with 5 seeds (30 seeds per treatment), while for cold conditions in soil, they were six replicates with 10 seeds (60 seeds for treatment). Six replicates were used during the determination of pigments and proline. All the results have been expressed as mean ± SE values.

## 3 Results

### 3.1 Seedlings in Drought Conditions

**Figure 1:**
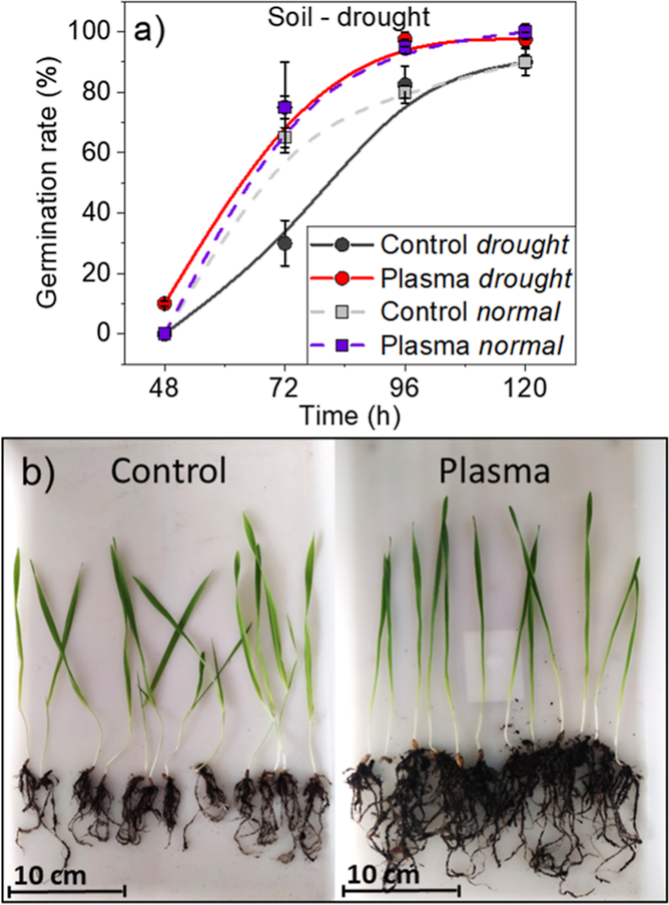
(a) Plots comparing the germination rate of control and plasma-treated seeds sown and grown in soil under drought and normal conditions. Data corresponding to germination rates in normal conditions are taken from ref (26). (b) Photograph of barley plants, including the roots, corresponding to control (left) and plasma-treated seeds (right) grown under drought conditions 10 days after seedling appearance.

Figure 1a shows that germination rates were slightly higher for plasma-treated than for control seeds for either drought or normal conditions. Thus, 72 h from sowing, 75% of plasma-treated seeds had already germinated, against 30 and 60% of control seeds for drought and normal conditions, respectively. Differences decreased but did not disappear 120 h after sowing; images in Figure 1b show that 10 days after sowing, the stalks developed in drought conditions reached a similar height for plasma-treated and control seeds (about 20 cm), although these stalks were slightly longer than those formed under normal conditions of sowing (about 17 cm, see the Supporting Information, Figure S3). Interestingly, in drought conditions, the radicular system was more robust and dense in plants grown from plasma-treated than control seeds (cf., Figure 1b). An evaluation of the root system (see Supporting Information Table S1) shows that the average number of roots, the main root length and diameter, and the average surface occupied by the roots are higher for the barley plants developed from plasma-treated seeds as compared with the control ones. Thus, the number of roots increased 20% whereas the main root length and diameter were found 70 and 80% larger upon plasma treatment after 10 days of seedling appearance in drought conditions.

**Figure 2:**
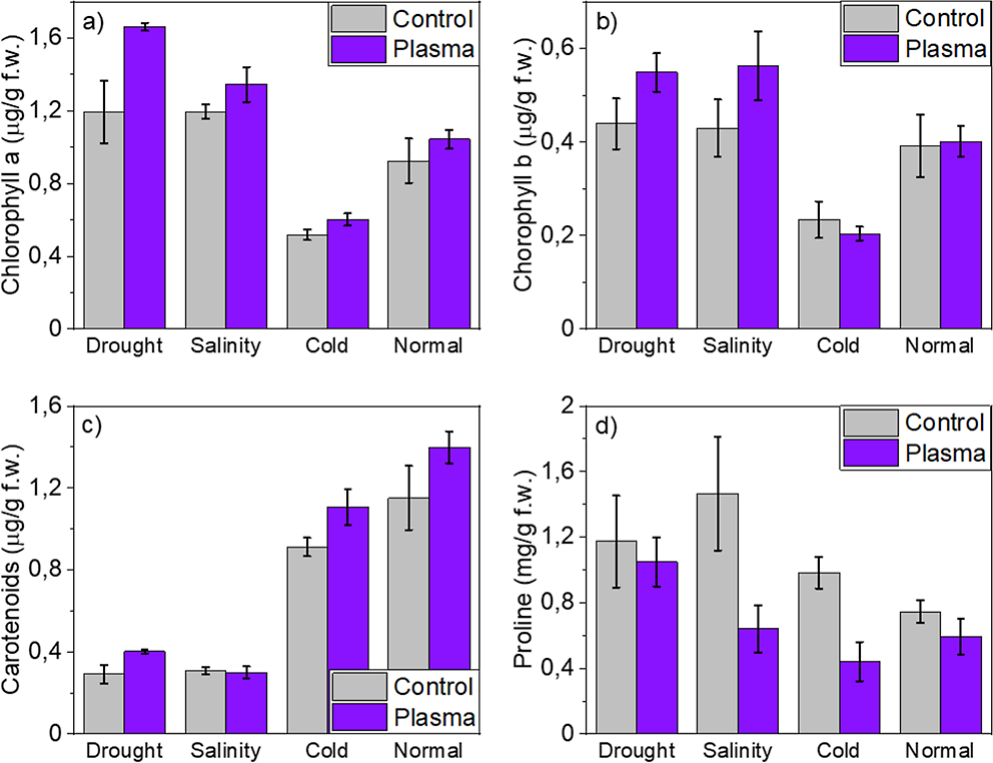
Pigments and proline concentrations in the leaves of plants developed from control and plasma-treated seeds in stress (drought, salinity, and cold) and normal conditions: (a) chlorophyll *a* pigment; (b) chlorophyll *b* pigment; (c) carotenoid pigment; and (d) proline amino acid.

Differences were also found in the concentration of biomarkers found in plants grown in either drought or normal conditions from plasma-treated and control seeds. Figure 2a,b shows that concentrations of chlorophylls *a* and *b* were higher in drought than in normal conditions. Figure 2c shows a reversal of this tendency with a drastic decrease in carotenoids for drought conditions, suggesting a compensation effect due to the scarcity of water. In all cases, a small but not negligible enrichment of pigments is observed comparing plants developed from plasma-treated vs control seeds (Figure 2a–c).

Figure 2d shows a significant decrease in proline concentration in plasma-treated vs control seeds for all conditions of sowing (i.e., a reversed tendency with respect to that found for pigments, though in much larger proportion). This general difference indicates that plasma-treated seeds are less proline demanding, even under stress conditions, and suggests that plasma treatment somehow releases the stress of young plants. In this way, plants seem to require less proline for an effective protection against membranes and protein degradation.^63–65^ In the particular case of drought conditions, the observed decrease was less important than in salinity or cold environments, although it was still clearly noticeable.

The differences found in germination rate, height of plants, size of root system, and concentration in leaves of pigments and proline biomarkers must be interconnected and demonstrate a beneficial effect of plasma treatment during the initial stages of plant growth. In other terms, these results prove a better adaptation of plasma-treated seeds for their use in moderate stress conditions, a tendency also observed for other seeds in recent studies in drought conditions.^38,39,66^

### 3.2 Seedlings in Salinity Conditions

**Figure 3:**
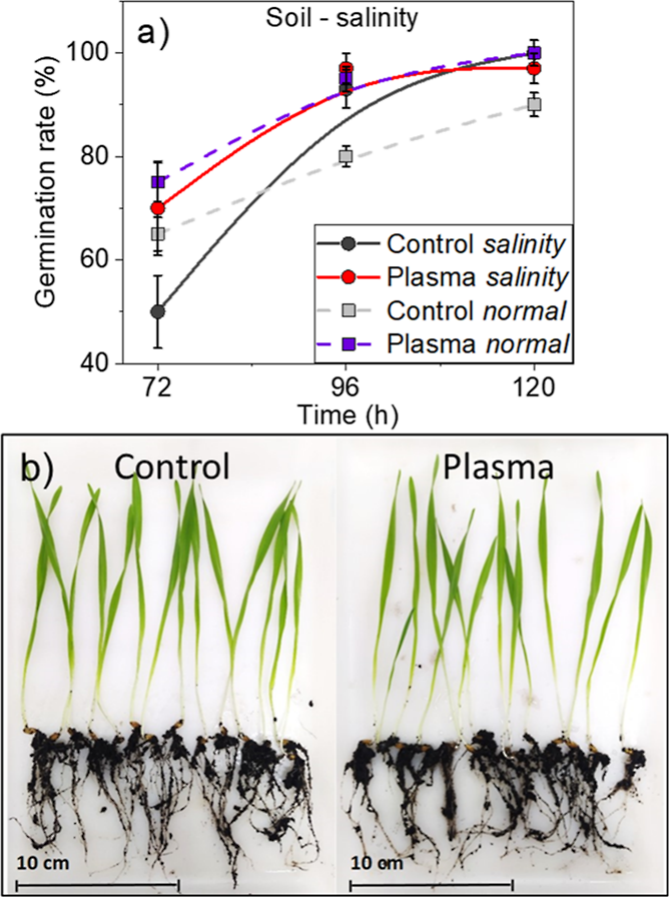
(a) Plots of the germination rate of control and plasma-treated seeds sown and grown in soil under salinity and normal conditions of irrigation. Data corresponding to germination rates in normal irrigation conditions are taken from ref (26). (b) Photographs of barley seed plants, including the roots, for control (left) and plasma-treated seeds (right) grown in salinity conditions 10 days after seedling appearance.

At the global scale, there is a progressive decrease in the fertility of soils subjected to salinization.^67^ Responding to this issue, in this work, we have investigated the germination rates and plant growth of plasma-treated seeds in salinity conditions. Germination rate results are shown in Figure 3a.

From the results in Figure 3a, it appears that salinity conditions produce a generalized initial decrease in germination rate, particularly for control seeds, although differences decrease 96 h after sowing. Similar differences in germination rate were observed for culture tests in Petri dish, where the number of germinated seeds for normal and salinity conditions was similar after 48 h/72 h (see Supporting Information Figure S4a). Meanwhile, plants developed from either control or plasma-treated seeds in normal and salinity conditions (15–16 cm) had similar sizes 10 days after seedling appearance, and their root system was also similar (see Figure 3b and Supporting Information Figure S3 and Table S2).

Interestingly, the differences in the concentrations of pigments and proline in the plants grown in salinity conditions were similar to those in drought conditions: more chlorophyll *a* and *b*, much less carotenoids, and more proline (cf., Figure 2). Similarly, concentrations of chlorophylls *a* and *b* were higher, that of carotenoids and, particularly proline, smaller in plants developed from plasma-treated seeds than in control seeds. These similar tendencies in biomarker concentrations during the initial stages of plant growth suggest that young plants respond similarly to these two types of stresses. However, it is remarkable that the significantly high decrease in the concentration of proline found in the plants evolved from plasma-treated seeds (proline concentration decreased by more than 50% for plasma-treated seeds with respect to control seeds under salinity conditions, Figure 2d). As in the drought case, it seems that young plants grown from plasma-treated seeds are much less stressed, also under salinity conditions.

Since the addition of exogenous proline to plants may compensate for the effect of water scarcity,^51–57^ we performed an experiment consisting of watering the seed simulating the salinity conditions but with an extra addition of proline (see the Materials and Methods section). We realized that 7 days after seedling appearance, plants were 35% shorter when adding proline (Figure S5). However, as reported in Supporting Information Figure S6, we also found that for both control and plasma-treated seeds, the concentration of pigments and proline in the leaves did not significantly differed whether plants were irrigated with proline incorporated in Milli-Q water or the saline solution. Differences were neither found for control or plasma-treated seeds. These results proved that addition of exogenous proline did not affect differently the final concentration of this amino acid in the leaves and that, therefore, changes observed in the plants developed from plasma-treated seeds (cf. Figure 2d) are due to endogenous metabolic effects induced by the plasma treatment.

### 3.3 Seedlings in Cold Conditions

**Figure 4:**
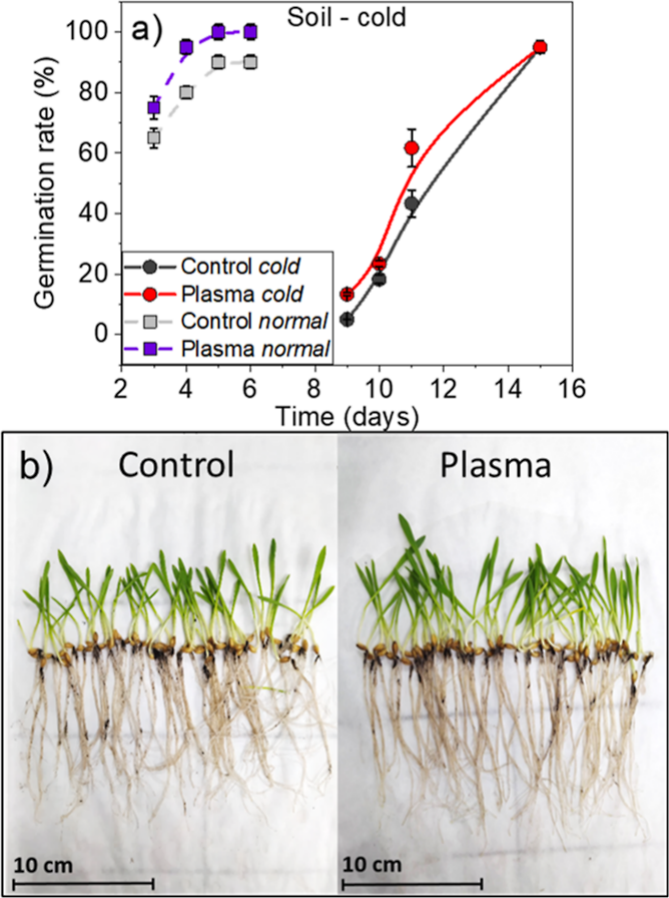
(a) Germination rate in soil at 5 and 24 °C of control and plasma-treated seeds. Data corresponding to the germination rates at 24 °C are taken from ref (26). (b) Photographs of plants evolved from control and plasma-treated seeds 15 days after sowing under cold conditions.

The germination of seeds is very sensitive to the ambient temperature. The influence of this parameter has been widely studied for warm conditions^68–71^ and less frequently for cold conditions.^72^ Dormancy periods and germination/growth cycles in seeds of natural vegetation are often controlled by the (low) temperature and humidity of the environment. In the current scenario of climate change, the survival of some natural species may be threatened by the lack of cold winters in certain regions.^73,74^ In this section, we report about the effect of low temperatures on the germination rate and initial stages of plant growth from control and plasma-treated seeds. Figure 4a reveals that the germination rate in soil of barley seeds is much slower at 5 °C than at 24 °C and that in the former case, seedlings only start to emerge from soil 9 days after sowing, against 3 days in the latter. However, it is noteworthy that also at low temperatures, the germination rate was higher for plasma-treated seeds, as clearly shown in Figure 4a 11 days after sowing. The general slowing down in the germination rate in cold conditions should be taken as an indication of a general decrease of metabolic activity of seeds/plants. This is also seen in the size of the stalks that, even 15 days after sowing, presented a height smaller than 4 cm (cf., Figure 4b). It is, however, noticeable that these plants presented very large roots (a rough estimation of the root/shoot ratio renders a value of 2, quite different to that found for the plants grown under salinity and drought conditions, 0.5 and 0.3, respectively). It is also relevant that the roots in plants evolved from plasma-treated seeds were slightly longer than those evolved for the control seeds, always keeping a similar root/shoot ratio (see an evaluation of the characteristics of the radicular system in Table S3 from the Supporting Information where a root length around 13.5 ± 0.3 cm was found for the plasma-treated seeds at cold conditions in comparison with the mean root length of 11.5 ± 0.3 cm found for the stressed control seeds).

Results in Petri dishes gathered in Figure S4b confirm that the germination rate is significantly slowed down at a low temperature of 20 °C. Indeed, at low temperatures, first seeds germinated only 72 h after sowing, while at normal temperatures, a significant portion of seeds had already germinated 24 h after sowing. Significantly, the germination rate of plasma-treated seeds in a Petri dish at low temperatures increased (ca. 40% at 72 h) in comparison with control seeds (ca. 25%).

The affectation of the growth metabolism at 5 °C is further supported when analyzing the concentration of pigments and proline in the leaves of plants grown in cold conditions. As shown in Figure 2, concentrations of chlorophylls *a* and *b* were quite small and that of carotenoids was high for cold conditions. Meanwhile, in comparison with control seeds, plasma treatment induced a small increase in chlorophyll *a* and carotenoids and a small decrease in chlorophyll *b*. Unlike this relatively small variation in the concentration of pigments between control and plasma-treated seeds, cold conditions produced a drastic decrease of more than 50% in the proline concentration in plants emerged from plasma-treated seeds with respect to control. As for the other stress conditions, this drastic decrease in proline concentration for the seedlings emerged at low temperatures further supports a release of metabolic stresses when seeds have been subjected to plasma treatments.

### 3.4 Water Uptake Capacity in Salinity and Cold Conditions

**Figure 5:**
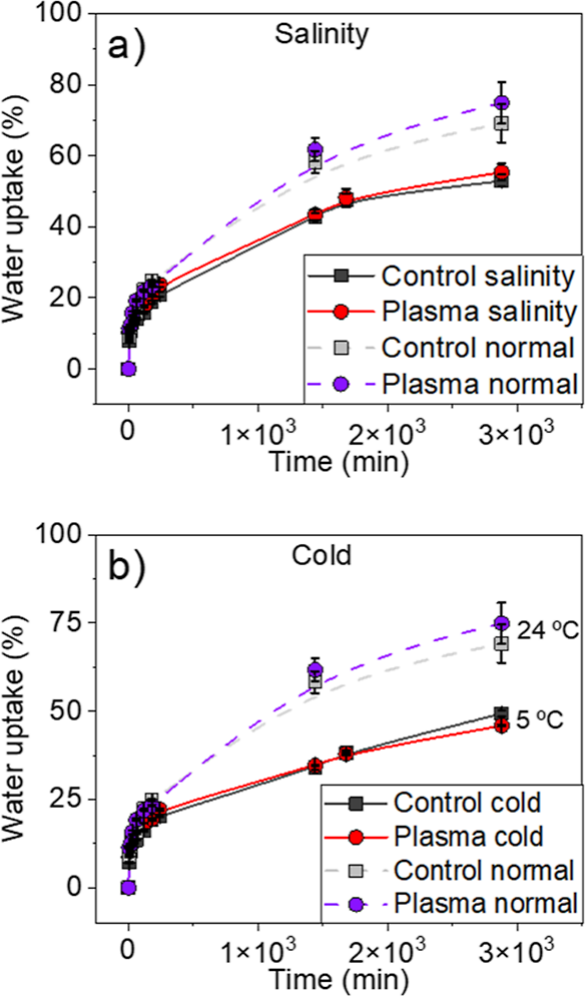
Water uptake capacity in salinity (a) and cold conditions (at 5 °C) (b), expressed as a percentage of the increase of weight of plasma-treated and control seeds. Results for an equivalent experiment with Milli-Q water at 24 °C (normal conditions) are included for comparison. Data corresponding to normal conditions are taken from ref (26).

Water imbibition capacity is an important control factor of germination and other metabolic functions.^75^ The similar tendencies in germination rates and biomarker concentrations found for drought and salinity conditions (cf. Figure 2) suggest that these stress conditions may affect germination rates and initial stages of plant growth in a similar way. Addressing this point, we have determined the water absorption capacity of seeds under salinity conditions (in soil drought conditions, the availability of water was imposed externally, and therefore, imbibition was not controlled by the seed capacity to absorb water but by the availability of this resource). Figure 5a shows that water imbibition capacity after 50 h decreased by 20–30% for the seeds immersed in a salt solution (0.2 M NaCl) in comparison with Milli-Q water (i.e., normal conditions). Data also showed that plasma-treated and control seeds took a practically equivalent amount of water in either condition. Therefore, this experiment proved that plasma treatment does not significantly affect the absorption capacity of seeds for either normal or salinity conditions.

Since the observed decrease in metabolic activity of the barley seeds/plants at 5 °C (cf., Figure 4) will likely induce a decrease in their imbibition capacity at this temperature, we have also analyzed whether the plasma treatment affects the water absorption capacity at 5 °C. Figure 5b shows an effective lowering in water imbibition of ca. 30–40% at 5 °C with respect to 24 °C, although, practically no differences were found between control and plasma-treated seeds.

## 4 Discussion

The previous results on the germination rate of barley seeds, plant growth, and phenotype characteristics have revealed significant differences for drought and salinity with respect to normal conditions (cf. Figures 1 and 3). Differences in germination rate and phenotype of young plants were even more significant for cold conditions, where long delays in germination, both in soil and a Petri dish, and a significant decrease in plant size were found (cf. Figure 4). A common observation was that water imbibition capacity significantly decreased under the investigated stress conditions (cf. Figure 5). Similar effects have been reported in previous works on the subject and various types of seeds.^28–31,37,42–47^

**Figure 6:**
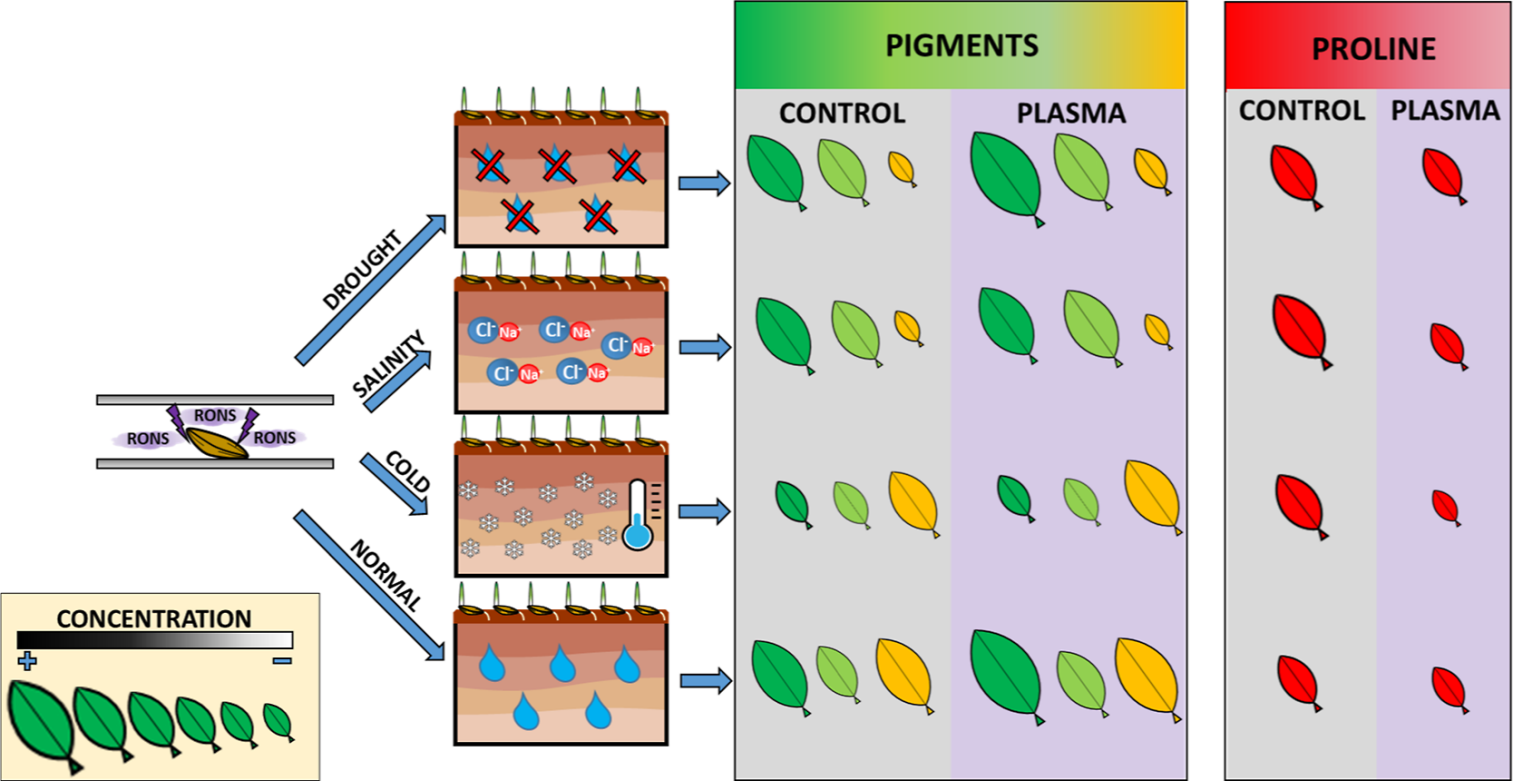
Schematic representation of pigments (chlorophyll *a* and *b* and carotenoids) and proline concentrations in the leaves of plants evolved from control and plasma-treated seeds sown in drought, salinity, and cold conditions. Normal conditions are included for comparison. Green leaves refer to chlorophylls *a* and *b*, orange leaves to carotenoids, and red leaves to proline.

In this paper, we have also determined the concentration in young plants of various pigments and the amino acid proline, taken as biomarkers of metabolism. Chlorophylls *a*, *b*, and carotenoid are responsible for the collection of light energy during the initial stages (i.e., photon capture) of the photosynthetic cycle. Each pigment covers a different wavelength range of the solar spectrum: 646–663 nm for chlorophylls *a* and *b* and 450–475 nm for carotenoids, plus quite intense bands for the three pigments in the UV region below 400 nm. The scheme in Figure 6 summarizes the tendencies found in the concentration of the selected biomarkers (chlorophylls *a* and *b*, carotenoids, and proline) in the leaves of plants developed from control and plasma-treated seeds in stress and normal conditions. In this image, the size of the leaves refers to concentration and the color to the type of pigment and proline.

In the next subsections, we discuss these changes in the light of similar experiments carried out for barley and other seeds, trying to highlight the beneficial effects of plasma treatments of seeds for their use in stress conditions.

### 4.1 Response of Barley Control Seeds to Stress Conditions

For control barley seeds, the tendencies schematized in Figure 6 do not exactly coincide with the tendencies reported for seeds of other plants sown in stress conditions. For example, Zhuang et al.^75^ reported a pronounced decrease of chlorophyll concentration in cucumber grown in drought conditions. According to Mafakheri et al.,^76^ drought produces a decrease in pigment concentration, whereas proline concentration increases notably. Similarly, Shin et al.^77^ reported that drought, salinity, and cold stresses affect watermelon plant growth parameters (number of leaves, height, shoot/root ratio) and produce a decrease in the chlorophyll *a* and *b* and an increase in proline contents. Drought also affects the carotenoid pigment concentration in eggplants^78^ or cotton.^79^ Our experiments with control barley seeds have revealed that chlorophylls *a* and *b* concentrations (cf. Figure 2) presented just a moderate decrease or no change for drought and salinity with respect to normal conditions. This behavior agrees with that found in salt-tolerant barley genotypes, which are marginally affected by salinity conditions and where chlorophyll *b* content may even increase^80^ due to physiological factors such as antioxidant potential, photosynthetic capacity, or ion uptake. It is also known that barley genotypes tolerant to water deficits^81^ present similar contents of chlorophyll in normal and drought conditions.^82^ Unlike chlorophylls, our experiments have shown that carotenoid concentration drastically decreased for drought/salinity vs normal conditions, suggesting a compensation mechanism between this and the chlorophyll pigments. Interestingly, in cold conditions, the general retardation of the metabolic activity of control barley plants (Figures 2 and 6) was related to a drastic decrease in the concentration of chlorophylls *a* and *b* that, to some extent, were substituted by carotenoids. This reversal in the concentration of pigments likely reflects an economy of resources in conditions imposing a low metabolic activity on the plants.

The amino acid proline is known to contribute to the adjustment of the osmotic equilibrium at the seed membranes, protecting them as well as proteins against dehydration. For this reason, proline tends to accumulate in plants of different species grown under drought and salinity stresses,^54,63–65,83^ an effect that coincides with the tendencies reported in Figures 2 and 6 for barley plants evolved from control seeds. In line with these previous works, we assume that the increase in proline in barley plants is a mitigation factor of drought/salinity stresses. According to Figures 2 and 6, an increase in the concentration of this amino acid is also found in barley plants for cold with respect to normal conditions. These results support that proline is a very sensitive biomarker to detect specific responses of young barley plants to the environment.

### 4.2 Plasma Treatment and Response to Stress Conditions

The experiments described in the Results section have shown that plasma treatments modulate positively the effects of stress conditions on the growth of plants: they compensate for germination delays (cf. Figures 1–3) and stimulate plant development (i.e., size) during the initial stages of growth (cf., Figures 1,3,4 and S3 and S6). Plasma treatments also modify the concentration of the selected biomarkers in the young plants compared to their respective controls (cf., Figures 2 and 6), particularly that of proline. Unlike earlier investigations on water imbibition in plasma-treated seeds, the results in Figure 5 have proved that plasma does not significantly alter the water imbibition capacity of seeds, in either normal or stress conditions. Therefore, the changes found in barley plants evolved from plasma-treated seeds must be attributed to metabolic modifications during the germination stages and initial phases of plant growth. These changes in metabolism give rise to changes in the concentration of the selected biomarkers. Summarizing the tendencies found in biomarker concentrations (cf. Figures 2 and 6), we can say that in drought and salinity conditions, plasma produces a moderate increase in the concentration of chlorophylls *a* and *b* with respect to normal conditions, while for cold conditions, it reinforces the tendency of substituting chlorophyll by carotenoid pigments, always to a relatively low pace. Most significant for all stressing conditions is that plasma induced large decreases in the concentration of proline, particularly for cold conditions, where the proline concentration reached a minimum value. We assume that plants emerging from plasma-treated seeds are less proline demanding because plasma triggers a series of biochemical processes that neglect the need of this amino acid in adverse media. The results of our experiment consisting of adding endogen proline under salinity conditions further support this conclusion.

Therefore, the reported changes confirm that plasma treatments induce modifications in the metabolism of barley seeds with beneficial effects that continue during the initial stages of plant growth. This evidence aligns with published results reporting that plasma treatment of barley seeds is beneficial for the evolved plants. For example, plasma can counterbalance the potassium deficit and the degradation of chlorophyll pigments experienced by the accumulation of sodium in plants grown in salinity conditions.^80,84^ It can improve the nutrient uptake capacity through the development of a robust root morphology^85^ (i.e., similar to our results in Figure 1 for drought conditions) or increase the activity of antioxidant enzymes and enhance the content of the pigments^18^ (i.e., similar to our results in Figure 2). Other works on barley seeds have reported about the influence of plasmas on the evolution of various biochemical markets and even DNA.^26,86–88^

The results reported here for barley plants are in line with recent studies with other plants proving that plasma gives rise to biochemical modifications. These have been attributed to the activation of certain enzymes, the modification of proteins, or the activation of specific growth and gene expression factors.^89–92^ A common hypothesis in these works on barley and other plants is that ROS and RNS plasma species formed on the surface of seeds may diffuse into their interior and affect key molecular mechanisms^21,22^ involving enzymes, proteins, and other biochemical markers (for a comprehensive account of these recent investigations, see the contribution of recent papers to the topic^10–14,20–22^). In a recent work from our group on barley seeds, we have shown that plasma affects the ABA (abscisic acid) growing factor in a similar way to hydrogen peroxide, both treatments leading to an increase in germination rates thanks to the enhanced ROS content.^26^ Herein, we have analyzed the content of ROS as indicated in the Materials and Methods section, trying to clarify the effect of the plasma treatment under stress conditions (see Figure S7 from the Supporting Information). We have found that peroxo-like species are more abundant in the stressed plasma-treated barley seeds compared to the nontreated ones, especially upon exposure to salinity and cold environments.

Without excluding the formation of other chemical species acting as ROS/RONS (evidence of the formation of NO_*x*_ species at the surface of seed was gathered by the X-ray photoemission analysis of seeds after plasma treatment^26^), a reasonable conclusion of this previous work is that peroxide or superoxide species formed during plasma activation of seeds produced a decrease in the ABA concentration in the plasma treated seeds. In this line, our results in the present work show that plasma also affects other biochemical markers as leaf pigments and proline and partially releases the negative effects of growing under stress conditions. We hypothesize that the ROS formed upon plasma treatment (cf. Figure S7) are also involved in the observed changes in biomarker concentrations. Since the water absorption capacity of seeds was practically unaffected by the plasma treatment, we assume that the main factor influencing the barley seed germination process is the triggering by the RONS of a series of complex metabolic processes that, affecting the production of the selected biomarkers and other specific biomolecules, contribute to improve the germination rate and phenotype characteristics of young plants.

The nature of these metabolic changes as well as the way in which they affect the phenotype of plants is complex and still a matter of debate. It is believed that the antioxidant capacity of specific enzymes,^14,18,24^ gene expression,^20,25^ or specific plant growth factors^24,35^ are affected by reaction with plasma-generated RONS. However, the exact nature and concatenation of metabolic reactions still require additional investigations considering specificities for each type of seeds and environmental conditions, as exemplified by the results in the present work.

## Supporting Information Available

*I*(*t*) and *V*(*t*) curves and Lissajous figure corresponding to the applied plasma treatment on barley seeds; the proline standard curve; the plant height after 10 days and the germination rate in Petri dishes and salinity or cold conditions in comparison with normal conditions for plasma-treated and control seeds, respectively; statistical analysis for the roots in drought and in salinity conditions, respectively; effect of exogenous proline in the height of the plants 7 days after sowing in salinity conditions as well as the effect of exogenous proline in pigments and endogenous proline concentration in plants grown in salinity conditions, respectively; statistical analysis for the roots in cold conditions; and ROS content (PDF)
