# Supplementary material for: Germination and First Stages of Growth in Drought, Salinity, and Cold Stress Conditions of Plasma-Treated Barley Seeds
Source: ACS Agric Sci Technol. 2023 Sep 6;3(9):760–70. doi: 10.1021/acsagscitech.3c00121 (PMC10520973; doi:10.1021/acsagscitech.3c00121)
Supplement: Supplementary file 1 — as3c00121_si_001.pdf [file as3c00121_si_001.pdf]

Germination and first stages of growth in drought, salinity and cold stress conditions of plasma treated barley seeds

Alvaro Perea-Brenes[1,2], Jose Luis Garcia[2], Manuel Cantos[2], Jose Cotrino[1,3], Agustín R. Gonzalez-Elipe[1], Ana Gomez-Ramirez[1,3*], Carmen Lopez-Santos[1,4 *]

[1]Nanotechnology on Surfaces and Plasma Laboratory, Institute of Materials Science of Seville, Consejo Superior de Investigaciones Científicas-Universidad de Sevilla, Seville, Spain

[2]Department of Plant Biotechnology, Institute of Natural Resources and Agrobiology of Seville, Consejo Superior de Investigaciones Científicas, Seville, Spain

[3]Departamento de Física Atómica, Molecular y Nuclear, Universidad de Sevilla, Seville, Spain

[4]Departamento de Física Aplicada I, Escuela Politécnica Superior, Universidad de Sevilla, Seville, Spain

* Correspondence:
Carmen Lopez-Santos; Ana Gomez-Ramirez
mclopez@icmse.csic.es; anamaria.gomez@icmse.csic.es

Supporting information

I(t) and V(t) curves and Lissajous figure corresponding to the applied plasma treatment on barley seeds

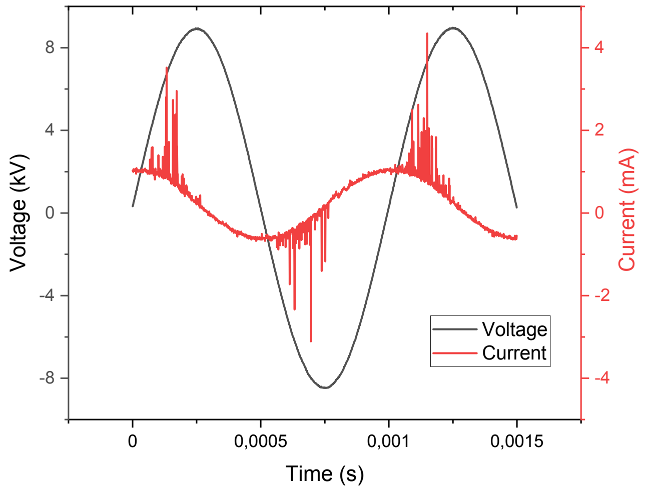

Fig. S1. (a) I(t) and V(t) curves and (b) Lissajous plot for the DBD reactor operated at 1kHz and 8.6 kV during seed treatments

Proline standard curve

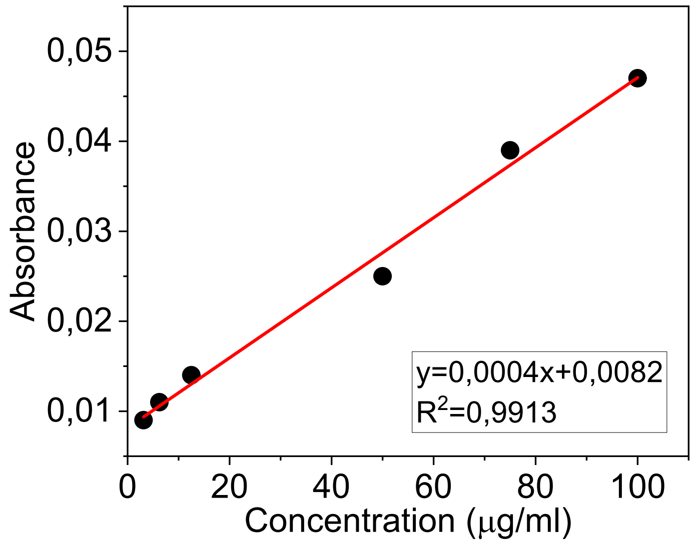

Fig. S2. Calibration curve of the proline solutions. This standard curve for absorbance correspond to samples diluted twenty times. Undiluted samples present a very high absorbance that may induce to errors.

Plant height

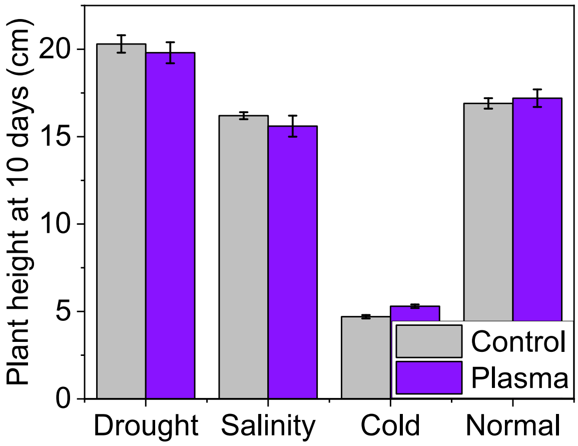

Fig. S3. Height of plants after ten days from sowing in substrate. Small differences were found between the heights of plants from treated and untreated seeds.

Statistical analysis for the roots in drought conditions.

Table S1.- Statistical analysis over 12 plants for the roots developed in young barley plants corresponding to control and plasma treated seeds sown in drought conditions after 10 days from seedling (total root surface around 72.6 cm^2 and 137.0 cm^2 for control and plasma treated seeds, respectively)

**seed**	**Roots / plant**		**Main root length (cm)**		**Main root diameter (cm)**		**Root average surface (cm^2)**	
	**control**	**plasma**	**control**	**plasma**	**control**	**plasma**	**control**	**plasma**
**1**	3	5	9,2	10,0	0,18	0,23	6,2	6,9
**2**	2	3	10,2	12,7	0,16	0,34	4,4	12,8
**3**	4	5	8,0	13,0	0,16	0,33	6,2	12,2
**4**	3	5	5,5	14,7	0,12	0,36	5,5	15,4
**5**	2	4	5,7	11,7	0,24	0,38	5,1	8,9
**6**	4	4	6,2	12,7	0,16	0,28	5,6	10,3
**7**	4	5	6,0	10,2	0,14	0,18	4,4	12,2
**8**	3	3	8,5	14,0	0,18	0,22	6,2	14,5
**9**	5	4	7,7	15,2	0,11	0,29	6,0	11,3
**10**	2	4	6,5	13,2	0,13	0,29	8,9	9,8
**11**	4	3	7,5	14,5	0,20	0,27	7,7	9,8
**12**	5	5	8,7	12,7	0,16	0,36	6,3	12,5
**mean**	**3,4**	**4,2**	**7,5**	**12,9**	**0,16**	**0,29**	**6,1**	**11,4**
**SD**	1,1	0,8	1,5	1,6	0,04	0,06	1,3	2,5
**SE**	**0,3**	**0,2**	**0,4**	**0,5**	**0,01**	**0,02**	**0,4**	**0,7**

Germination rate in Petri dishes and *salinity* or *cold* conditions in comparison with *normal* conditions for plasma treated and control seeds.

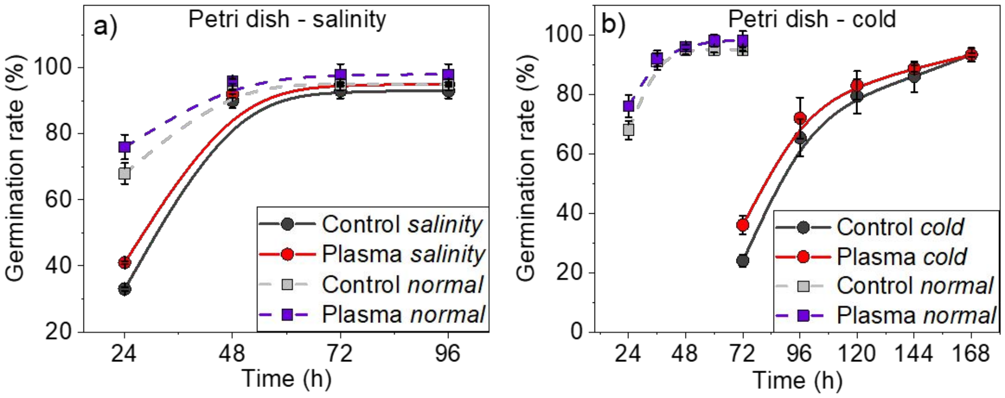

Fig. S4. Germination rate of untreated and plasma treated barley seeds in Petri dish under *salinity* (a) and *cold* (b) conditions compared to *normal*.

Statistical analysis for the roots in salinity conditions.

Table S2.- Statistical analysis over 14 plants for the roots developed in young barley plants corresponding to control and plasma treated seeds sown in salinity conditions after 10 days from seedling (total root surface around 40.4 cm^2 and 47.2 cm^2 for control and plasma treated seeds, respectively)

seed	Roots / plant		Main root length (cm)		Main root diameter (cm)		Root average surface (cm^2)	
	control	plasma	control	plasma	control	plasma	control	plasma
**1**	2	4	10,5	8,6	0,28	0,19	4,2	5,3
**2**	2	2	7,5	4,2	0,16	0,17	4,0	1,3
**3**	3	3	9,4	10,0	0,18	0,24	3,5	3,4
**4**	4	4	7,9	8,4	0,32	0,11	4,2	3,2
**5**	2	3	7,9	8,4	0,17	0,21	1,6	4,8
**6**	3	3	10,5	6,7	0,20	0,24	3,4	2,3
**7**	4	4	8,0	9,9	0,14	0,24	1,9	3,7
**8**	3	3	8,7	10,8	0,14	0,28	1,9	3,3
**9**	2	5	8,6	6,9	0,07	0,19	2,2	4,1
**10**	3	2	10,5	6,5	0,16	0,22	3,8	3,1
**11**	5	3	8,2	9,5	0,11	0,18	3,7	2,7
**12**	3	4	8,5	7,2	0,21	0,26	2,5	3,2
**13**	3	3	8,3	7,4	0,20	0,21	0,7	2,0
**14**	3	3	9,8	9,6	0,19	0,29	2,8	4,7
**mean**	**3,0**	**3,3**	**8,9**	**8,2**	**0,18**	**0,22**	**2,9**	**3,4**
**SD**	0,9	0,8	1,1	1,8	0,06	0,05	1,1	1,1
**SE**	**0,2**	**0,2**	**0,3**	**0,5**	**0,02**	**0,01**	**0,3**	**0,3**

Effect of exogenous Proline in the height of the plants after seven days from sowing in salinity condition

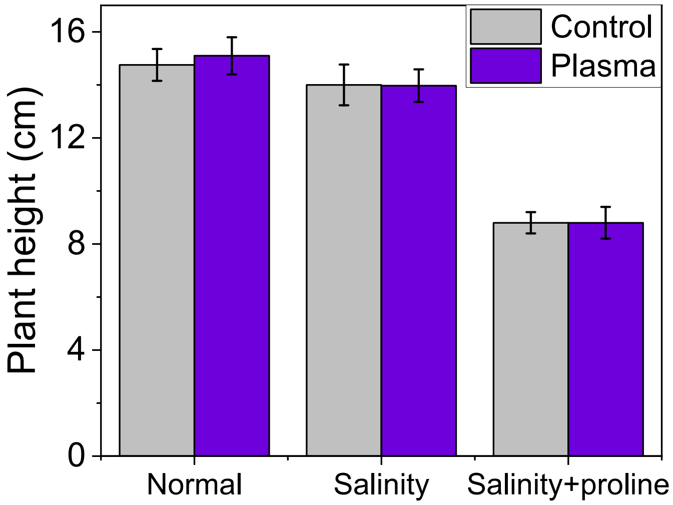

Fig. S5. Height of the plant from pristine and plasma treated seeds under salinity stress (Salinity), salinity stress with exogenous proline (10 mM) application (salinity+proline) and normal conditions (normal). Proline addition decreased plant height under salinity conditions.

Effect of exogenous Proline in pigments and endogenous proline concentration in plants grown in *salinity* conditions.

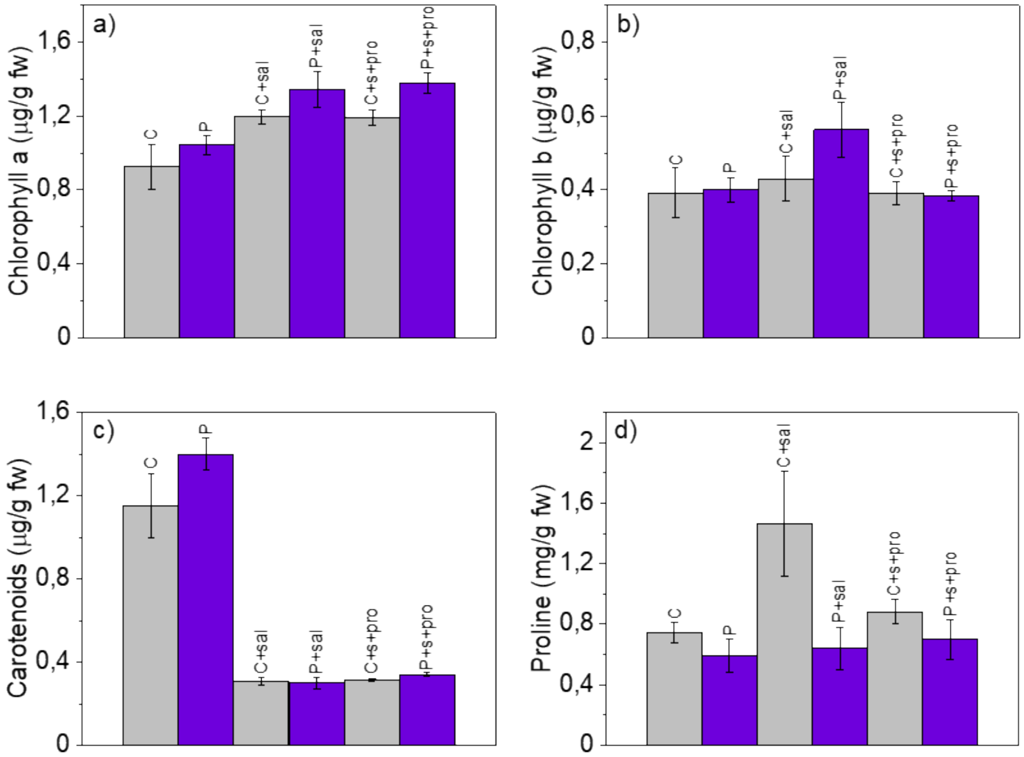

C=control C+sal=control in salinity C+s+pro= control in salinity with proline
P=plasma P+sal=plasma in salinity P+s+pro= plasma in salinity with proline

Fig. S6. Pigments and proline concentrations in the leaves of plants grown from pristine and plasma treated seeds under salinity stress (control+sal and plasma+sal), salinity stress with exogenous proline (10 mM) application (control+sal+pro and plasma+sal+pro) and normal conditions (nc). a) Chlorophyll a pigment. b) Chlorophyll b pigment. c) Carotenoid pigment. d) Proline aminoacid.

Statistical analysis for the roots in cold conditions.

Table S3.- Statistical analysis over 40 plants for the roots developed in young barley plants corresponding to control and plasma treated seeds sown in cold conditions after 15 days from seedling (total root surface around 85.4 cm^2 and 104.8 cm^2 for control and plasma treated seeds, respectively)

**seed**	**Roots / plant**		**Main root length (cm)**		**Main root diameter (cm)**		**Root average surface (cm^2)**	
	**control**	**plasma**	**control**	**plasma**	**control**	**plasma**	**control**	**plasma**
**1**	3	3	8,3	13,1	0,02	0,01	1,4	3,7
**2**	2	3	8,7	12,9	0,02	0,02	1,5	4,3
**3**	3	3	11,3	13,4	0,02	0,02	1,8	1,7
**4**	3	2	10,1	14,1	0,02	0,02	1,6	1,5
**5**	3	3	9,9	9,2	0,02	0,01	2,1	1,5
**6**	3	4	11,6	11,9	0,01	0,02	1,1	2,9
**7**	3	3	9,3	12,0	0,01	0,02	1,9	3,0
**8**	2	3	10,2	11,8	0,01	0,02	2,8	2,0
**9**	4	2	12,2	12,0	0,01	0,01	2,1	1,9
**10**	3	3	10,2	14,0	0,01	0,01	1,6	5,4
**11**	2	3	11,8	12,1	0,01	0,01	2,1	1,6
**12**	3	2	12,0	13,6	0,01	0,01	1,4	3,4
**13**	2	2	13,0	14,6	0,02	0,01	1,7	3,1
**14**	3	3	10,4	11,5	0,01	0,01	2,6	3,3
**15**	2	3	12,7	14,2	0,02	0,01	2,5	3,5
**16**	3	3	9,6	14,0	0,02	0,01	2,3	4,8
**17**	3	3	9,0	15,0	0,01	0,01	2,5	2,1
**18**	3	4	13,6	10,9	0,01	0,02	2,0	2,2
**19**	3	3	12,3	13,0	0,01	0,01	1,5	2,3
**20**	2	4	10,7	14,0	0,01	0,01	1,3	1,5
**21**	3	3	10,0	12,6	0,01	0,01	2,5	3,2
**22**	3	3	11,3	14,3	0,01	0,02	2,9	1,8
**23**	3	3	11,2	18,0	0,01	0,02	1,5	2,8
**24**	4	3	12,3	13,8	0,01	0,02	2,3	1,7
**25**	3	4	14,0	13,5	0,01	0,02	2,7	1,1
**26**	3	3	12,9	14,4	0,01	0,02	2,6	1,2
**27**	3	3	14,7	15,1	0,02	0,02	4,0	2,7
**28**	2	3	12,8	13,6	0,02	0,02	3,1	2,3
**29**	3	4	13,8	13,3	0,02	0,02	1,4	4,8
**30**	3	3	13,1	11,7	0,02	0,02	1,2	3,2
**31**	3	2	12,0	14,3	0,01	0,02	3,1	2,1
**32**	2	2	11,1	14,8	0,02	0,02	1,9	2,6
**33**	4	3	14,2	15,9	0,02	0,01	2,8	2,5
**34**	3	3	8,1	18,8	0,02	0,02	1,7	2,1
**35**	3	4	11,3	13,7	0,01	0,01	2,3	2,4

**36**	3	3	10,5	14,9	0,01	0,02	1,5	1,9
**37**	2	3	11,3	11,2	0,02	0,02	2,6	2,9
**38**	3	2	14,6	13,7	0,01	0,01	3,7	4,0
**39**	3	3	12,3	12,3	0,02	0,01	2,3	2,0
**40**	4	3	12,5	14,3	0,01	0,02	2,0	2,0
**mean**	**2,9**	**3,0**	**11,5**	**13,5**	**0,01**	**0,01**	**2,1**	**2,6**
**SD**	0,6	0,6	1,7	1,8	0,00	0,00	0,7	1,0
**SE**	**0,1**	**0,1**	**0,3**	**0,3**	**0,00**	**0,00**	**0,1**	**0,2**

ROS content

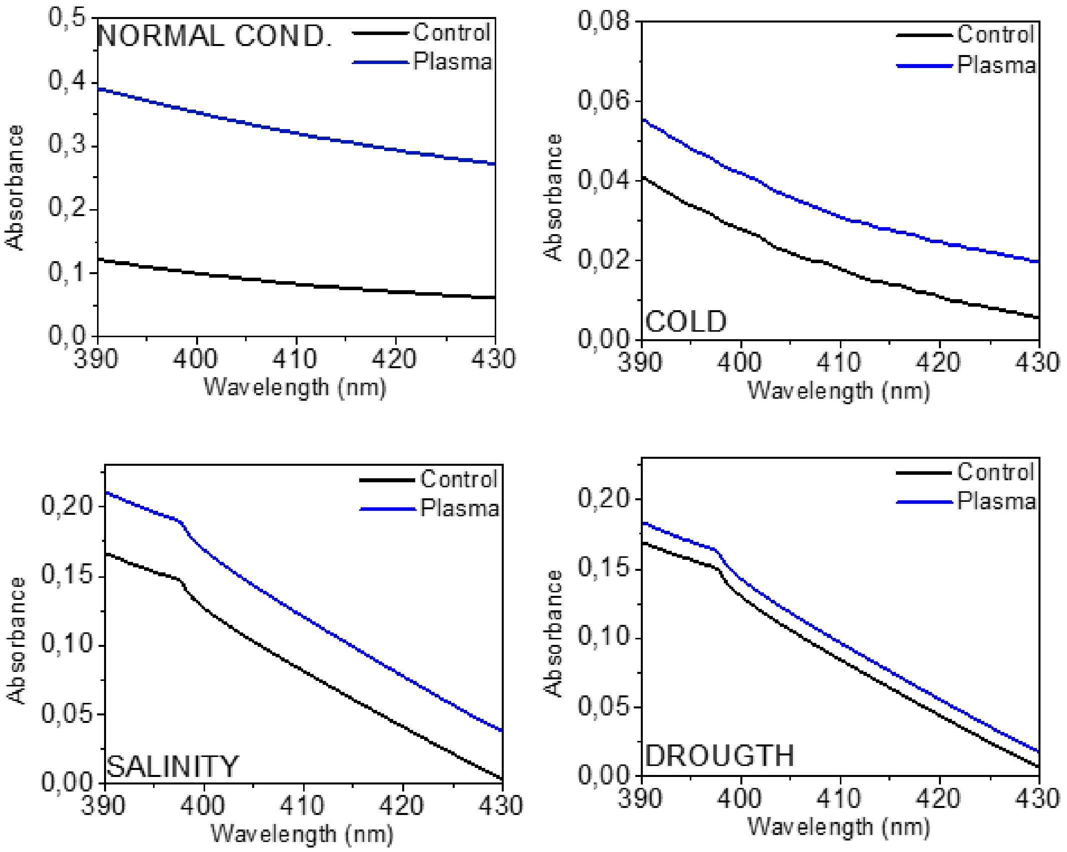

Fig. S7. Absorbance spectra at a wavelength range around 410nm of the liquid resulting for the soaking, grinding and first centrifugation of seeds after adding titanium (III) sulphate as described in the Materials and Methods section to determine the relative content of peroxo-like, H_2O_2 species for control and plasma treated seeds at normal conditions and after being exposed to a short simulation of *drought*, *salinity* and *cold* stress.
